# Improving Care for Older Adults with Cancer in Canada: A Call to Action

**DOI:** 10.3390/curroncol31070279

**Published:** 2024-06-30

**Authors:** Sarah Cook, Shabbir Alibhai, Rajin Mehta, Marie-France Savard, Caroline Mariano, Dominique LeBlanc, Danielle Desautels, Rossanna Pezo, Xiaofu Zhu, Karen A. Gelmon, Tina Hsu

**Affiliations:** 1 Tom Baker Cancer Centre, University of Calgary, Calgary, AB T2N 4N2, Canada; 2 Department of Medicine, University Health Network, University of Toronto, Toronto, ON M5G 2C4, Canada; 3 Sunnybrook Health Sciences Centre, University of Toronto, Toronto, ON M4N 3M5, Canada; 4 The Ottawa Hospital Cancer Centre, University of Ottawa, Ottawa, ON K1H 8L6, Canada; 5 BC Cancer Vancouver Centre, University of British Columbia, Vancouver, BC V5Z 4E6, Canada; 6 Centre Hospitalier Universitaire de Québec, Université Laval, Québec, QC G1V 0A6, Canada; 7 CancerCare Manitoba, University of Manitoba, Winnipeg, MB R3E 0V9, Canada; 8 Cross Cancer Institute, University of Alberta, Edmonton, AB T6G 1Z2, Canada

**Keywords:** geriatric oncology, geriatric assessment, older adults, cancer, systemic therapy, toxicity

## Abstract

Most patients diagnosed with and dying from cancer in Canada are older adults, with aging contributing to the large projected growth in cancer incidence. Older adults with cancer have unique needs, and on a global scale increasing efforts have been made to address recognized gaps in their cancer care. However, in Canada, geriatric oncology remains a new and developing field. There is increasing recognition of the value of geriatric oncology and there is a growing number of healthcare providers interested in developing the field. While there is an increasing number of dedicated programs in geriatric oncology, they remain limited overall. Developing novel methods to delivery geriatric care in the oncology setting and improving visibility is important. Formal incorporation of a geriatric oncology curriculum into training is critical to both improve knowledge and demonstrate its value to healthcare providers. Although a robust group of dedicated researchers exist, increased collaboration is needed to capitalize on existing expertise. Dedicated funding is critical to promoting clinical programs, research, and training new clinicians and leaders in the field. By addressing challenges and capitalizing on opportunities for improvement, Canada can better meet the unique needs of its aging population with cancer and ultimately improve their outcomes.

## 1. Introduction

The aging population accounts for a large, predicted increase in cancer incidence in Canada [1]. However, advancements in the care of this group have lagged due to slow progress in clinical care, research, and education of providers. Older adults, aged 65 and over, account for most patients diagnosed with, and dying from, cancer [2]. Older adults face inequities in all aspects of their cancer journey from screening to diagnosis, and treatment to survivorship and end of life (EoL) care. The current system, however, is ill equipped to provide optimal care to this population, which can have increased vulnerability to stressors. Research advances suggest that geriatric assessment (GA) and management can help identify vulnerabilities, which when addressed can improve patient-related outcomes [3,4,5,6]. Despite this, development of services to help operationalize these findings is lagging. Current healthcare providers receive little training in caring for older adults. In addition, older patients are woefully under-represented in the clinical studies that form the basis of treatment recommendations. Combined, these challenges hinder improvements in care for older adults with cancer in Canada. Here, we present a synthesis of the state of geriatric oncology in Canada. This is compiled using the authors’ knowledge of published and unpublished initiatives as leaders in the field within Canada. The intent of this paper is to identify gaps and challenges in the Canadian landscape and propose a dedicated strategy to accelerate developments in clinical care, research, and education about older adults with cancer, which is imperative to improve care for this large segment of the cancer population.

## 2. Healthcare Inequities and Barriers to Care in Older Adults with Cancer

The aging of the Canadian population is the main driver for the rising cancer incidence. This is unsurprising, as the hallmarks of aging and cancer overlap, reflecting the same critical underlying process, i.e., accumulating cellular damage [7]. The Canadian Cancer Society (CCS) projects that two-thirds of all cancers diagnosed will be in older adults aged 65 years. Older adults therefore comprise most Canadians living with and dying from cancer [2]. This has stark implications for all healthcare workers, as older adults present unique challenges across the cancer care spectrum.

**Figure 1:**
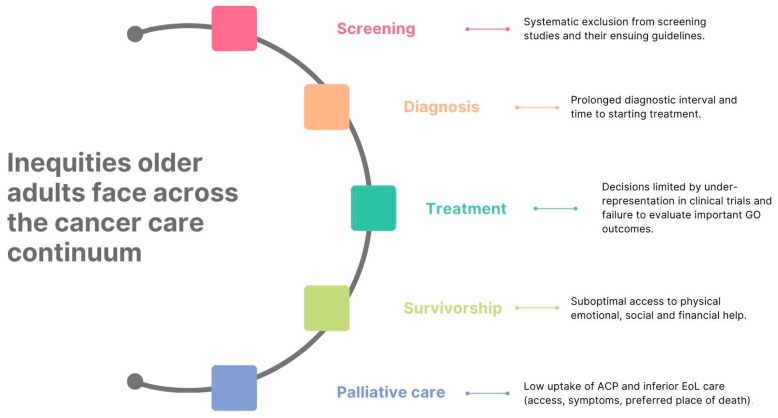
Summary of the inequities in cancer care of older adults in Canada. ACP = advanced care planning, EoL = end of life, and GO = geriatric oncology.

While significant survival gains from cancer have been made over the past few decades, these improvements have not been equally distributed, with large gaps seen between younger and older adults ≥65 [8]. Over three-quarters of cancer deaths are in Canadians ≥65 years of age and for most adult cancers, survival rates fall with advancing age [2]. Although this may reflect limitations in life expectancy as patients age, inequities older adults face from screening to diagnosis and treatment to survivorship and/or palliative care may also contribute, as summarized in Figure 1 [9,10].

Older adults are less likely to be screened for cancer, a disparity that increases with age, potentially contributing to worse survival outcomes [11]. Older adults are less likely to be included in screening studies and without supporting evidence for screening, it is unsurprising that they are often systematically excluded from resulting guidelines. This particularly impacts adults ≥75 years of age [12,13,14]. Screening patients based on age, as is largely the case in Canada, requires careful consideration, as aging is heterogenous, and chronologic age does not always reflect biologic age. Health and life expectancy can vary drastically among older adults.

Older adults in Canada are at risk of experiencing longer delays until diagnosis and are less likely to be biopsied [15,16]. Diagnostic delays result in a series of negative sequalae including higher stage at presentation, more intensive treatment needs resulting in greater toxicity, and ultimately worse survival [17,18,19,20,21,22,23,24]. Older Canadians can experience further delays in treatment initiation, contributing again to cancer care inequities [25]. Evidence suggests these age-related inequalities may have widened during the COVID-19 pandemic [26,27].

From a treatment perspective, older adults remain under-represented in research studies despite representing most patients diagnosed [28]. Furthermore, those older adults who are included in studies are often highly selected, very fit patients with fewer comorbidities, and thus may not represent the average older adult seen in clinic. This may lead clinicians to be concerned about applicability of data to older, more vulnerable patients seen in clinic, where little information about the rates of toxicity in this population is available. Undertreatment may therefore result. Patient care and decision making may also be hindered by the fact that outcomes important to patients, such as the effects of treatment on function and cognition, are often not collected [29,30]. Treatment decisions may also be impacted by physician biases, relating to ageism and presumptions about patients’ desires for treatment and/or its utility [9,10]. Treatment decisions can also be influenced by patient concerns regarding transportation, financial costs, and “inconveniencing” family and friends with treatment visits, worries not always shared with their healthcare team [30].

Survivorship relates to the long-term physical, mental, emotional, social, and financial sequalae of a cancer diagnosis and treatment. In older adults, survivorship may be further complicated by chronic physical impairment that can threaten functional independence, cognitive decline with impacts on maintaining autonomy, and psychosocial concerns relating to depression and difficulties reintegrating into their community, which is essential for social well-being [31]. Although many older Canadian cancer survivors may report physical, emotional, and practical needs, over half may be unable to obtain the required help [32]. Areas highlighted as requiring improvements in survivorship care provided to older adults here in Canada relate to service delivery (particularly pertaining to side effect management), relationships (including increased support for self and caregivers), and practical assistance (notably with activities of daily living and finances) [33].

Palliative care, as recommended by the ASCO, should be received by all advanced cancer patients, early in their disease course, but delivery in older adults is complex [34]. Many of the validated tools used for symptom assessment are not adapted for older adults, with a heavy reliance on self-reporting and a strong focus on physical symptoms rather than physical functioning [35]. Where symptoms are identified, palliation is complicated by geriatric syndromes, comorbidities, and polypharmacy, with mindful prescribing and non-pharmacologic strategies needed [36]. Uptake of advanced care planning in older cancer patients is low, with provider, caregiver, and patient barriers contributing [37,38,39,40]. Being older is also a risk factor for suboptimal EoL care as it pertains to access, managing terminal symptoms (pain, breathlessness, delirium), discussions around death and dying, preferred place of death, and hospice utilization [36,41,42].

Given older adults represent most cancer patients seen in Canadian clinical practice, addressing these existing inequities, and optimizing their care, is crucial.

## 3. Improving Outcomes in Older Adults with Cancer—The Role of Geriatric Assessment

Older adults are a diverse population whose cancer care is often more challenging due to concomitant comorbidities, geriatric syndromes, polypharmacy, heterogenous life expectancy, and differing treatment preference, much of which is not commonly captured in routine oncologic assessment [43]. These vulnerabilities complicate oncology-directed treatment decisions, particularly systemic therapy. This can lead to both “over”- or “under”-treatment, with less fit patients receiving cancer treatment with a low likelihood of benefit and fit patients not receiving cancer treatments with a high likelihood of benefit. Both treatment decisions compromise outcomes [8,44,45].

Much attention has therefore been paid to GA. GA is a multidimensional process that seeks to evaluate a patient’s health in multiple domains, including medical, psychosocial, and functional, to identify vulnerabilities not otherwise captured through traditional oncologic assessment. While assessment is important, it is often the intervention and management that is important to capitalize on outcomes. This process of addressing identified impairments is often referred to as GA and management (GAM) or comprehensive geriatric assessment (CGA).

GA can predict several important outcomes including chemotherapy toxicity, early discontinuation of chemotherapy, functional decline, and early mortality [46,47,48,49]. It can also change oncologic treatment decisions for one in four patients [50]. Several phase III randomized controlled trial (RCT) data comparing GAM to usual oncology care have now been completed (Table 1). We present an overview of the most commonly cited and pivotal studies, with an assessment of quality (Table 2), to provider readers with an understanding of major findings supporting the use of GAM in oncology in order to provide context for the paper and recommendations. It is not meant to be a comprehensive systematic review. The authors refer interested readers to the following reviews which include additional studies conducted in this area [50,51,52]. Important proven outcomes include a reduction in moderate to severe (grade 3+) toxicity in patients receiving systemic therapy (predominantly chemotherapy) and improved rates of chemotherapy completion [3,4,5]. One study suggested GAM improved QOL in patients receiving systemic therapy [6]. However, another did not [53]. The effect of GAM on healthcare utilization was also mixed [6,53]. Importantly, no survival differences were seen in any study.

Considering this data, several international organizations, including the International Society of Geriatric Oncology (SIOG) and the American Society of Clinical Oncology (ASCO) recommend GAM in patients aged ≥65 contemplating systemic therapy [54,55]. While GA is very adaptable and can be administered in a variety of ways (e.g., self-administered, electronically, in clinic) and within different healthcare contexts (e.g., through a geriatrician, multidisciplinary team, or within the oncology clinic), uptake of GA has been poor [56]. Cited barriers include lack of knowledge on how to perform a GA, resource limitations, and lack of time [56]. The ASCO therefore updated their guidelines in 2023 to try and address these barriers, unveiling the Practical Geriatric Assessment (PGA) tool, a structured GA which prioritizes evaluation of key geriatric domains associated with cancer care outcomes, namely physical/cognitive/emotional health, comorbidities, polypharmacy, nutrition, and social support. The PGA additionally estimates chemotherapy toxicity, recognizing that validated toxicity tools for targeted treatments and immunotherapy remain lacking. For each domain in the PGA, the ASCO advise assessment measures that can be efficiently completed in clinic and provide suggestions about how to utilize findings and address vulnerabilities identified (management), an important part of improving patient outcomes and actualizing the benefits of GAM reported in randomized studies [51]. The use of PGA was a consensus recommendation by the ASCO panel (Type: Informal consensus; Evidence quality: Moderate; Strength of recommendation: Weak).

**Table 1:** Summary of pivotal RCTs evaluating the impact of GA and GA-driven interventions.

Study	Population	Intervention	Comparison	Outcome Measures	Significant Results
GAIN [3] N = 605	- Age ≥65 - Planned for chemotherapy ± targeted tx. 1 US center	GA, SPICES, and CARG-TT. GA assessed domains of functional status, comorbidity, cognitive/psychological state, social activity/support, and nutritional status. SPICES evaluated common geriatric syndromes: sleep disorders, problems with eating/feeding, incontinence, confusion, evidence of falls, and skin breakdown. Results reviewed and interventions directed by geriatric trained MDT.	Results reviewed by oncologist.	1°: Gr ≥ 3 tx toxicity. 2°: - Chemo dose modifications and/or d/c. - ACP completion. - Healthcare utilization. - OS.	↓ Gr ≥ 3 tx toxicity in intervention arm (50.5% vs. 60.6%, *p* = 0.02). ↑ in ACP completion in intervention arm (28.4% vs. 13.3%, *p* < 0.001).
GAP70+ [4] N = 718	- Age ≥70 - Stage III/IV - Planned for tx with high risk of toxicity - ≥1 GA domain impairment. Multiple US centers	GA assessing domains of physical performance, functional status, comorbidity, cognition, nutrition, social support, polypharmacy, and psychological status. GA summary and recommended interventions developed by study team for oncologist review.	No GA summary or recommendations provided to oncologist.	1°: Gr 3–5 tx toxicity. 2°: - Tx intensity. - OS.	↓ Gr 3–5 tx toxicity in intervention arm (51% vs. 71%, aRR 0.74, *p* = 0.0001). ↑ likelihood of reduced tx intensity (aRR 1.38, *p* = 0.015).
GERICO [5] N = 142	- Age ≥70 - Stage II-IV colorectal cancer - Planned for adjuvant or 1st line palliative chemo - Life expectancy ≥3 mo - ECOG 0–2 - Vulnerability identified using G8 screening tool. 2 Danish centers	GA assessing domains of co-morbidity, psycho-cognition, nutrition, and functional and physical status. Results reviewed and interventions directed by study team.	SOC by oncology team.	1°: Chemo completion with no additional dose reductions or delays (although oxaliplatin excluded). 2°: - Chemo dose reductions and/or delays. - AEs. - DFS. - PFS. - OS. - Colorectal cancer mortality.	↑ chemo completion without additional dose reductions or delays in intervention arm (45% vs. 28%, *p* = 0.04). Difference most prominent with adjuvant chemo (*p* = 0.01) versus palliative (*p* = 0.75). ↓ subsequent dose reductions in intervention arm (28% vs. 45%, *p* = 0.04).
INTEGERATE [6] N = 154	-Age ≥70 - Planned for systemic tx. 3 Australian centers	GA assessing domains of co-morbidities, medications, physical/cognitive/psychological social functioning, frailty, falls, nutrition, sensory impairment, immunization status, ACP, and chemo toxicity risk. Results reviewed and interventions directed by dual trained GO during serial visits.	SOC by oncology team.	1°: Change in hrQoL. 2°: - Functional status. - Mood. - Nutrition. - Anticancer tx modification. - Healthcare utilization. - Institutionalization. - OS.	↓ decline in hrQoL with intervention (overall main effect *p* = 0.039, effect size = 0.38). ↑ ED presentations (multivariable-adjusted incidence RR 0.59, *p* = 0.005), unplanned hospitalizations (multivariable-adjusted incidence RR 0.60, *p* = 0.007), and unplanned hospital days (multivariable-adjusted incidence RR 0.77, *p* < 0.0001).
5C [53] N = 350	- Age ≥70 - Referred for 1st or 2nd line adjuvant or palliative systemic tx. -Life expectancy >6 mo - ECOG 0–2. 8 Canadian centers.	GA assessing domains of functional status, cognition, nutrition, medications, co-morbidities, mobility, and falls. Results shared with oncologist. Results reviewed and interventions directed by team of GO fellows, a geriatrician, and a nurse.	SOC by oncology team.	1°: QoL. 2°: - Functional limitations. - Gr 3–5 tx toxicity and/or d/c - Tx modification. - OS.	No significant difference in any 1° or 2°. outcome.
Dumontier et al. [57]. N = 160	- Age ≥75 -Hematologic malignancy -not eligible for transplantation -initial consultation with hematologist-oncologist -Frail and pre-frail patients 1 US center	Consultation by a geriatrician. GA included assessment of function, falls, comorbidity, polypharmacy, cognition, mood, and nutrition.	Standard of care	1°: OS at 1 year. 2°: -unplanned healthcare utilization within 6 months (ED visits, unplanned hospitalization admissions, days in hospital). -documented end-of-life goals of care discussions.	No difference in survival at 1 year (18.3% vs. 21%, *p* = 0.65). Increased odds of EOL goals-of-care discussions (OR 3.12). No difference in ED visits, hospital admissions or duration of hospital stay.

Abbreviations: AE = adverse event; aRR = adjusted risk ratio; CARG-TT = Cancer and Aging Research Group chemotherapy toxicity tool; chemo = chemotherapy; d/c = discontinuation; DFS = disease-free survival; ECOG = Eastern Cooperative Oncology Group; ED = Emergency Department; GA = geriatric assessment; GO = geriatric oncologist; Gr = grade; hrQoL = health-related quality of life; MDT = multidisciplinary team; mo = months; OR = odds ratio; OS = overall survival; PFS = progression-free survival; QoL = quality of life; RR = rate ratio; SOC = standard of care; tx = treatment; US = United States; 1° = primary; 2° = secondary; ↓ = decreased; and ↑ = increased.

**Table 2:** Study quality assessment of pivotal RCTs evaluating the impact of GA and GA-driven interventions.

Trial	Adequate Randomization	Concealed Allocation	Sufficient Sample Size	Similar Groups	Double Blinded	Validated and Reliable Measures	Adequate Follow Up	ITT Analysis	Overall Potential Risk of Bias ✩
GAIN [3]	√	√	√	√	×	√	√	√	Low
GAP70+ [4]	√	√	√	×	×	√	√	?	Low–moderate
GERICO [5]	√	?	√	√	×	√	√	√	Low–moderate
INTEGERATE [6]	√	√	√	√	×	√	√	√	Low
5C [53]	√	√	√	√	×	√	√	√	Low
Dumontier et al. [57]	√	?	√	√	×	√	√	√	Low–moderate

Note: √ indicates criteria were met; × indicates criteria were not met; ? indicates insufficient detail, not reported, and/or uncertain if criteria were met. ✩ Ratings are based on the estimation of whether the criterion was met and extent of potential bias, not simply on reporting. Abbreviations: ITT, intention to treat.

## 4. Current State of Geriatric Oncology in Canada

Geriatric oncology (GO) is a new and developing field. In some countries, such as the United States and France, GO is more firmly established with formal training programs, focused GO clinical programs, and a robust GO presence in clinical trials. In Canada, however, the field of GO is less well established. Below, we outline the current state of GO in Canada.

### 4.1. Clinical Care

Currently, there are only five specialized GO clinical programs, all concentrated within Eastern Canada. Three are in Quebec (Montreal, Sherbrooke, and Levis) and two in Ontario (both in Toronto) [58]. A previously identified clinic in British Columbia has closed. A recent initiative funded by a pharmaceutical company to connect clinicians who care for older adults with breast cancer has also resulted in discussions to establish more formalized connections between oncologists and geriatricians in Alberta and in Sault Ste. Marie.

Montreal has the most established program, which has been operational for more than 15 years [58]. Patient volume varies, with two of the GO programs reporting that they see 100–300 new consults annually, compared to fewer than 50 new consults annually at the other three centres. Fitness for treatment, multimorbidity, and cognition are the most common reasons for consultation. Between one and four physicians are involved in each clinic. Access to allied health professionals is highly variable. Some programs are well supported but most have limited or no multidisciplinary support. Beyond these specialized clinics, it is difficult to capture if and how GO is being delivered during routine oncology visits. A survey of Canadian healthcare professionals working in medical, radiation, and surgical oncology found one in four respondents screen for frailty in their daily practice, but the use of a formal screening tool was uncommon, as was the use of the Cancer and Aging Research Group chemotherapy toxicity risk calculator. Commonly cited reasons for not doing so included lack of knowledge about the tools, inadequate resources to follow-up on screening results, and time constraints. Collaboration between oncology and geriatrics was also uncommon, predominantly due to poor access to or availability of geriatricians, and to a lesser extent lack of time and knowledge [59].

### 4.2. Research

Several Canadian clinicians/researchers have dedicated programs of research studying issues pertaining to older adults with cancer. This research ranges from prospective studies in GA to systematic reviews/scoping reviews on GO topics to economic analyses to qualitative work assessing how older adults make decisions [60,61,62,63,64,65]. A highlight of Canadian contributions to the field includes the 5C RCT (described in Table 1) comparing GA and management with usual oncologic care. This study represented one of the first research collaborations resulting from the Canadian Network on Aging and Cancer (CNAC), a network of healthcare professionals interested in improving care for older adults with cancer through clinical, research, and educational initiatives [53].

In addition to this, an increasing number of clinicians and researchers have sought to answer questions related to the clinical care of older adults with cancer, such as why they are undertreated [66,67]. As older adults are often excluded from clinical studies, particularly therapeutic studies, new strategies to target older adults with cancer such as pragmatic, simplified study designs are being used to try to address this. An example is the REaCT-70 study (currently actively recruiting), evaluating the harms and benefits of endocrine therapy in patients 70 years and older, with lower risk breast cancer [68].

### 4.3. Education

Despite the growing proportion of patients seen who are older, there is no established curriculum in GO within oncology training in Canada. A recently conducted study suggests that more than three quarters of medical oncology trainees receive some exposure to GO, though the majority receive between 1 and 5 h of formal training over the course of two years of training [69]. Most of this training (59.6%) is in the form of clinical teaching. Data also suggest more than half of Canadian radiation oncology trainees lack confidence in managing geriatric issues such as comorbidities, polypharmacy, and functional and cognitive impairments, with 73% reporting that additional training in these areas would be helpful [70].

Unlike in the United States, there is no established pathway to dual geriatrics and oncology certification in Canada. A growing number of clinicians, however, have pursued additional training in GO, many of whom now lead the development of the field in Canada. In Canada, there is a GO fellowship at McGill University which has trained six clinicians (geriatricians and a radiation oncologist) but the lack of established funding for interested candidates remains an important barrier. There is also now a fellowship program at the Princess Margaret Cancer Centre, supported by philanthropy, which has trained eight medical oncologists and geriatricians, with an additional two currently enrolled. Additional trainees have completed Informal (ad hoc) fellowship programs in other parts of Canada, such as at the Ottawa Regional Cancer Centre and the Sunnybrook Cancer Centre.

Gaps in GO knowledge and skills also exist in practicing Canadian healthcare professionals. A CNAC-led survey of predominantly physicians and nurses found respondents lacked confidence in issues relating to mental health, polypharmacy, GO models of care, and helping patients recover function post-treatment [59]. Educational workshops and meetings for Canadian healthcare professionals are emerging, including an annual Canadian GO conference, organized by the CNAC, with the fourth held in 2023 in Ottawa. The Canadian Association of Nurses in Oncology (CANO) has an established special interest group in GO which has held several workshops and webinars. In addition, several specialty organizations, including the Canadian Association of Medical Oncologists (CAMO), Canadian Association of General Practitioners in Oncology (CAGPO), and Canadian Association of Pharmacy in Oncology (CAPhO) have also included GO topics at their annual conferences.

## 5. Challenges, Opportunities and a Call to Action to Improve Care for Older Adults

Older adults with cancer in Canada continue to face challenges in accessing equitable healthcare as it relates to screening, diagnosis, treatment, survivorship, and palliative care. Despite phase III data supporting the benefits of GA, implementation within routine oncology practice remains challenging, and access to specialized GO clinical services remains limited in Canada. Advancing the field of GO has been further hindered by the research and educational challenges previously described.

There are, however, opportunities to improve GO within the Canadian healthcare system. There is a growing number of newly trained oncologists and geriatricians interested in this field. Many have pursued additional GO training and within their faculty positions, and are helping to advance the care of older adults with cancer in Canada. As Canada’s population continues to age, bringing with it a rise in cancer incidence, now more than ever, attention must be paid to GO. The authors make a call to action to improve the care we provide to this vulnerable patient population (Table 3).

Improving outcomes for older adults with cancer will require a shift in oncology culture and attitude. Although many clinicians recognize that this population has unique needs, some clinicians believe they “already take care of older adults” and do not recognize the value of geriatric-oncology-specific care. To many, GO remains a “niche” specialty, inhibiting its uptake and integration into oncology culture [71]. Some clinicians may also harbor negative biases with respect to perceived benefits of treating older adults. The “very old” (≥80 years) are particularly vulnerable as they are less likely to be investigated, referred, and treated [11]. These attitudes must change.

To accelerate progress, more formal recognition and acceptance of the value of geriatric care within the field of oncology is needed. This comes from education of healthcare providers about the benefits of GA and/or geriatric input within the context of cancer care, as well as better and more timely access to specialists with expertise in geriatrics and/or GO. Training nurses to screen older patients to identify those who may benefit from a formal GA can also help to sensitize clinicians on an ongoing basis to considering geriatric-related concepts in patient care, as well as give patients access to some sort of GA. Establishment of GO services is also important. Developing such services would encourage collaboration, giving oncologists the opportunity to interact with geriatricians and directly experience the benefits of co-managing older cancer patients, including bidirectional learning and establishing a common understanding and language about caring for this patient population. In some cases, beginning with a collaborative model allows motivated oncologists to learn to address GO issues without direct ongoing support from geriatricians.

Education of the workforce is important to help accelerate improvements in the care of older adults with cancer. While clinicians with a dedicated interest in GO is important, the reality is that the growth of this patient population will outstrip their capacity to provide care and the care of older adults with cancer will remain under the purview of cancer specialists and their teams. To touch most older adults affected by cancer, and to improve their care, training of the greater oncology workforce is essential. Formal recognition and incorporation of GO training into Canadian oncology residency training by governing bodies, such as the Royal College of Physicians and Surgeons of Canada, is thus essential to ensure delivery of curricula to trainees in various programs, as well as demonstrating to trainees the importance of this topic within their profession.

Expanding research programs in GO in Canada is important to stimulate advances in the field and ultimately improve care of older adults with cancer. Development of a critical mass of researchers in the field is important to cultivate a variety of research skillsets and expertise upon which effective collaboration can occur. This can also provide mentorship and inspire young researchers to become interested in the field. Development of a network to connect researchers who work in different centers can help promote collaboration, reduce duplication of work, and accelerate advancements in the field. Formal collaborations with established research entities such as the Canadian Cancer Trials Group and the Cancer and Aging Research Group would be advantageous. While work remains to be done in multiple facets of GO, research in implementation sciences to look at alternate methods to implement GA (including nursing-led GA, patient self-reported GA, and systematic screening) would be timely and impactful, particularly due to increased demands on oncologists due to more effective and tolerable cancer treatment options available.

Funding is critical to accelerating progress in all the aforementioned areas. Clinicians are increasingly interested in providing GO services but establishing programs has been limited by financial constraints. Recognition by the government of the value of such programs through the allocation of funding would help accelerate the development of GO services, allowing older adults in a variety of locales greater access to the benefits of GA. The result of a recent economic analysis suggests that GO clinics can save the government healthcare dollars (more than CAD 7000 per patient assessed) [62]. Dedicated calls for research related to older adults with cancer, by organizations such as the Canadian Cancer Society and the Canadian Institutes of Health Research, would convey value to researchers and encourage them to engage in this field. Lastly, dedicated and consistent funding for fellowships in GO would help train clinicians who can develop and implement GO services and help to lead the development of the field in Canada.

In addition to increased funding, the authors call for a purposeful and coordinated plan to engage cancer care organizations and politicians involved in healthcare administration. This is important in advancing the care of older adults with cancer in Canada. As an initial step, a study of the current state of affairs in geriatric oncology in Canada would be helpful, including formal characterization of available clinical programs and current outcomes in older adults with cancer. Subsequently sharing evidence of improvements from interventions on outcomes that positively impact patients but also society at large would be beneficial. This plan must involve collaboration with older adults and their caregivers who can advocate for perceived priorities but also provide real stories and faces to the unique problems facing this patient population.

## 6. Conclusions

In summary, although older adults with cancer comprise an increasing proportion of patients diagnosed with and who die of cancer, advances in their care have been slow and there remain barriers to them receiving optimal caring. Data support that GA and management improves outcomes for patients. Given the already aging population, there is an urgency to implement measures that improve clinical care and outcomes for these patients, educate providers about their needs, and accelerate research, including how best to operationalize the benefits of GA into clinical care. While there is already increasing recognition of this populations’ needs, recognition and establishment of more clinical GO programs, dedicated funding, and encouragement of research in the field is needed to help accelerate current efforts to address the needs of this aging population and ultimately to improve their outcomes.

## Figures and Tables

**Table 3:** Summary of challenges and opportunities to accelerate development of GO in Canada.

**Challenges**
Lack of recognition by clinicians of benefits of GO beyond current oncologic care
Clinician biases and nihilism resulting in inequities in management of older adults with cancer
Paucity of data to guide ideal management of older adults with cancer due to under-representation in studies
Lack of training in caring for older adults with cancer
Lack of dedicated funding supporting initiatives in GO (clinical, research, and educational)
**Opportunities**
Growing interested in GO with more clinicians pursuing additional training in GO
Growing cadre of clinicians trained in GO to lead development of GO programs, research, and training
**Call to Action**
*Improve access to GO and integrate it into oncology culture*
Change attitudes of clinicians towards older patients and towards value of GO
Educate clinicians about the added value of GA and GO
Increase the availability and visibility of GO in oncology -Train nurses to screen older adults with cancer with geriatric screening tools -Encourage and support the development of GO services through policy and dedicated funding
Consider novel methods of delivering and integrating GA into clinical care
Engage with cancer care organizations and politicians to develop a coordinated strategy to advance care of older adults with cancer in Canada
*Education*
Incorporate GO training into training programs for oncology trainees and allied healthcare professionals working with cancer patients. Lobby for GO curricula to be formally recognized and included in oncology training (including exams)
Establish dedicated funding supporting training for trainees interested in pursuing additional training in GO
*Research*
Stimulate research and the development of research programs in GO through funding specifically earmarked for research in this population
Foster collaborations between GO researchers and established national networks to increase study opportunities for older adults with cancer
Encourage research into implementation sciences to help develop novel methods to integrate GA into clinical care
